# Posterior Quadrants and Malleolar Perforations as Key Predictors of Hearing Loss in Chronic Otitis Media: A Clinicoradiological Correlation

**DOI:** 10.7759/cureus.100015

**Published:** 2025-12-24

**Authors:** Zohda Tayyaba, Zeeshan Ahmad, Sana Ansari, Abdur Rahman, Raihan Mannan, Kamlesh Chandra

**Affiliations:** 1 Otolaryngology - Head and Neck Surgery, Jawaharlal Nehru Medical College and Hospital, Aligarh Muslim University, Aligarh, IND; 2 Physiology, All India Institute of Medical Sciences, Patna, Patna, IND

**Keywords:** chronic otitis media, conductive hearing loss, malleolar perforation, mastoid pneumatization, posterior perforation, tympanic membrane perforation

## Abstract

Introduction

Chronic otitis media (COM) of the mucosal type, characterized by a central tympanic membrane (TM) perforation, is a common cause of preventable conductive hearing loss in developing countries. While the impact of perforation size on hearing loss is established, the independent and combined roles of perforation site, malleolar involvement, and mastoid pneumatization remain underexplored. The primary objective of the study is to assess the relationship between hearing loss and the size and site of TM perforation. The secondary objective is to assess the effect of the duration of discharge and mastoid bone pneumatization on hearing loss.

Methods

This prospective observational study was conducted over a period of two years, during which a total of 210 ears from 161 patients with inactive mucosal COM were evaluated. TM perforations were categorized by size (small ≤4 mm, medium 4-8 mm, large >8 mm) and site (anterosuperior (AS), anteroinferior (AI), posterosuperior (PS), posteroinferior (PI), umbo). Pure-tone audiometry was used to measure the air-bone gap (ABG), and radiological assessments (Schuller’s view) were used to evaluate mastoid pneumatization. Statistical analysis included analysis of variance (ANOVA), t-tests, chi-square tests, and two-way ANOVA.

Results

Larger perforations, and those involving posterior quadrants, showed significantly greater ABGs (p < 0.001). Among single-quadrant perforations, PS (23.9 ± 7.6 dB) and PI (19.3 ± 7.6 dB) had the highest hearing loss. Four-quadrant perforations had the greatest ABG (32.47 ± 9.04 dB). Malleolar perforations were associated with higher ABG (29.26 ± 9.4 dB) than non-malleolar perforations (18.25 ± 8.7 dB). Two-way ANOVA revealed site as a stronger predictor of hearing loss than size. Duration of discharge and reduced mastoid pneumatization further exacerbated hearing loss.

Conclusion

Posterior site and larger size of TM perforations independently contribute to significant hearing loss. Incorporating perforation characteristics and radiological findings can improve diagnostic precision, guide surgical planning, and enhance patient outcomes in COM.

## Introduction

Chronic otitis media (COM) remains a global health concern, especially in low- and middle-income countries, where overcrowding, poor sanitation, and limited access to healthcare persist [1]. Defined by the World Health Organization (WHO) as a chronic inflammation of the middle ear and mastoid cavity lasting more than two months with persistent otorrhea through a perforated tympanic membrane (TM), COM is one of the leading causes of acquired hearing impairment worldwide [2].

The TM plays a vital role in auditory mechanics by transmitting acoustic energy from the external auditory canal to the ossicular chain within the middle ear. Any disruption of this membrane, particularly through perforation, compromises its vibratory capacity and reduces the efficiency of sound conduction [3].

Consequently, understanding how the size and location of the perforation affect hearing loss is of paramount clinical importance, especially in tailoring surgical interventions such as tympanoplasty. This research, therefore, seeks to systematically assess the association between the size and site of TM perforation and the degree of conductive hearing loss in patients with mucosal-type COM, using a quadrant-based approach and validated audiometric tools. The secondary objective of the study is to establish a relationship between the duration of hearing loss and mastoid bone pneumatization (using appropriate radiographic assessment) and the degree of hearing loss.

## Materials and methods

Study design and setting

This was a prospective observational study, conducted over two years (Oct 2019-Oct 2021), in the Department of Otolaryngology - Head and Neck Surgery at a tertiary care hospital in North India. The Institutional Ethics Committee, under the National Ethics Committee Registry for Biomedical and Health Research, issued approval for this study (no. 09/FM/1257; dated October 23, 2019).

Study population

Participants were recruited from the outpatient and inpatient departments during the study period, and informed consent was obtained from all individuals before inclusion. Patients of both sexes, aged 15 years and above, were included if they presented with a dry central perforation confined to the pars tensa of the TM. Patients were excluded if their clinical examination revealed cholesteatoma, marginal perforation, tympanosclerotic patch, or evidence of healed otitis media. Other exclusion criteria included the presence of sensorineural hearing loss, eustachian tube dysfunction, history of topical or systemic ototoxic drug exposure, and complaints of tinnitus or vertigo. Eustachian tube function was assessed clinically by Valsalva and Toynbee maneuvers. These exclusion criteria were applied to ensure a homogeneous study population with inactive mucosal disease and purely conductive hearing loss.

Clinical evaluation

A comprehensive clinical evaluation was undertaken for all enrolled patients. Detailed history was recorded, including chief complaints, duration of symptoms, and relevant demographic and clinical information. Otoscopic examination was performed using the Welch Allyn MacroView 3.5V Halogen HPX Otoscope (Welch Allyn, Inc., Skaneateles Falls, NY, USA), and findings were subsequently confirmed through otoendoscopic evaluation. To eliminate inter-observer variability and maintain consistency, all examinations were performed by the principal investigator.

Classification of perforation

TM perforations were categorized based on their size and location. Perforation size was measured and classified as small (≤4 mm), medium (>4 mm but <8 mm), and large (≥8 mm) - subjective evaluation from endoscopic pictures. The location of the perforation was identified using quadrant-based anatomical demarcation and included the anterosuperior (AS), anteroinferior (AI), posterosuperior (PS), and posteroinferior (PI) quadrants of the pars tensa. Cases involving multiple quadrants were also noted. Perforations encroaching on the umbo were categorized as malleolar, and the rest as non-malleolar.

Radiological evaluation

Preoperative radiological evaluation comprised plain X-ray of the mastoid in Schuller's view (antero-posterior projection), performed using standard digital radiography. Images were systematically assessed by a blinded radiologist for mastoid air cell opacification (complete/partial), cortical bone sclerosis (increased density/thickening), and decalcification. Sclerosis was recorded as present if increased radiopacity involved >50% of the mastoid cortex. Of note, only 177 ears (84% of the cohort) were available for X-ray due to socio-economic constraints.

Audiological assessment

Assessment of hearing loss was initially conducted using Gardner tuning forks at frequencies of 256 Hz, 512 Hz, and 1024 Hz. This was followed by a formal audiometric evaluation in a sound-attenuated room using the MAICO MA42 clinical audiometer with TDH-50P earphones. The degree of conductive hearing loss was quantified by calculating the air-bone gap (ABG) across standard frequencies.

Statistical analysis

All collected data were compiled and analyzed using standard statistical software. Quantitative variables, such as age and ABG, were expressed as mean ± standard deviation (SD). Categorical variables, including sex, perforation size category, and site of perforation, were summarized using frequencies and percentages. To assess the association between perforation size and site and the degree of hearing loss, one-way analysis of variance (ANOVA) was employed. When a statistically significant difference was observed, post-hoc pairwise comparisons were conducted using the Bonferroni correction to control for the risk of Type I error due to multiple comparisons. For comparisons between two independent groups, such as malleolar versus non-malleolar perforations, the Student’s independent sample t-test was used. In addition, a two-way ANOVA was performed to evaluate the independent and interaction effects of perforation size and site on ABG values. This analysis allowed for assessment of whether the relationship between perforation size and hearing loss varied depending on the location of the perforation, and vice versa. The interaction term in this model tested whether the combined effect of size and site differed from their individual effects. To examine the correlation between the duration of ear discharge and the degree of hearing loss, Spearman’s rank correlation coefficient (ρ) was calculated, given the non-parametric nature of duration categories. Chi-square tests were employed to analyze the association between categorical variables, such as radiological findings and perforation size categories. All statistical analyses were two-tailed, and a p-value of <0.05 was considered statistically significant throughout the study.

## Results

Demographic and clinical characteristics

In this study, 161 patients were recruited, and a total of 210 ears were examined. The age group of patients in the study varied from 15 to 55 years, with the highest proportion in the 15-25 years category. Among all the ears examined, 128 were female, while only 82 ears were from male patients. Among the 161 patients enrolled, 49 patients (30%) had bilateral involvement. Regarding the geographic distribution, 59% (124 ears) of the study population belonged to rural areas, and 41% (86 ears) were from urban settings. Chief complaints of patients in our study group included ear discharge for varying periods, observed in 95.2% (200 ears). Hearing loss was the second most frequent complaint, reported in 57.6% (121 ears), highlighting the significant functional impact of TM perforation in this population. Other less commonly reported symptoms included ear pain, present in 7.14% (15 ears), and tinnitus, documented in 4.28% (nine ears) of the cases. This symptom distribution emphasizes that, while discharge and hearing impairment are predominant manifestations, otalgia and tinnitus may also be present in a smaller subset of patients with inactive mucosal COM.

Perforation characteristics

The size of the TM perforation significantly influenced the ABG (p < 0.001, ANOVA). Larger perforations were associated with higher ABGs, with small perforations exhibiting the least hearing loss, and large perforations showing the most significant impairment (Table 1).

**Table 1:** Effect of perforation size on hearing loss (ABG) ABG: air-bone gap; ANOVA: analysis of variance

Size of perforation	Total no. of ears	Percentage	Mean ABG (dB)	p-value
Small	40	19%	13.67 ± 9.4	<0.001 (ANOVA)
Medium	99	47%	22.13 ± 7.1	
Large	71	34%	32.47 ± 8.8	

**Figure 1:**
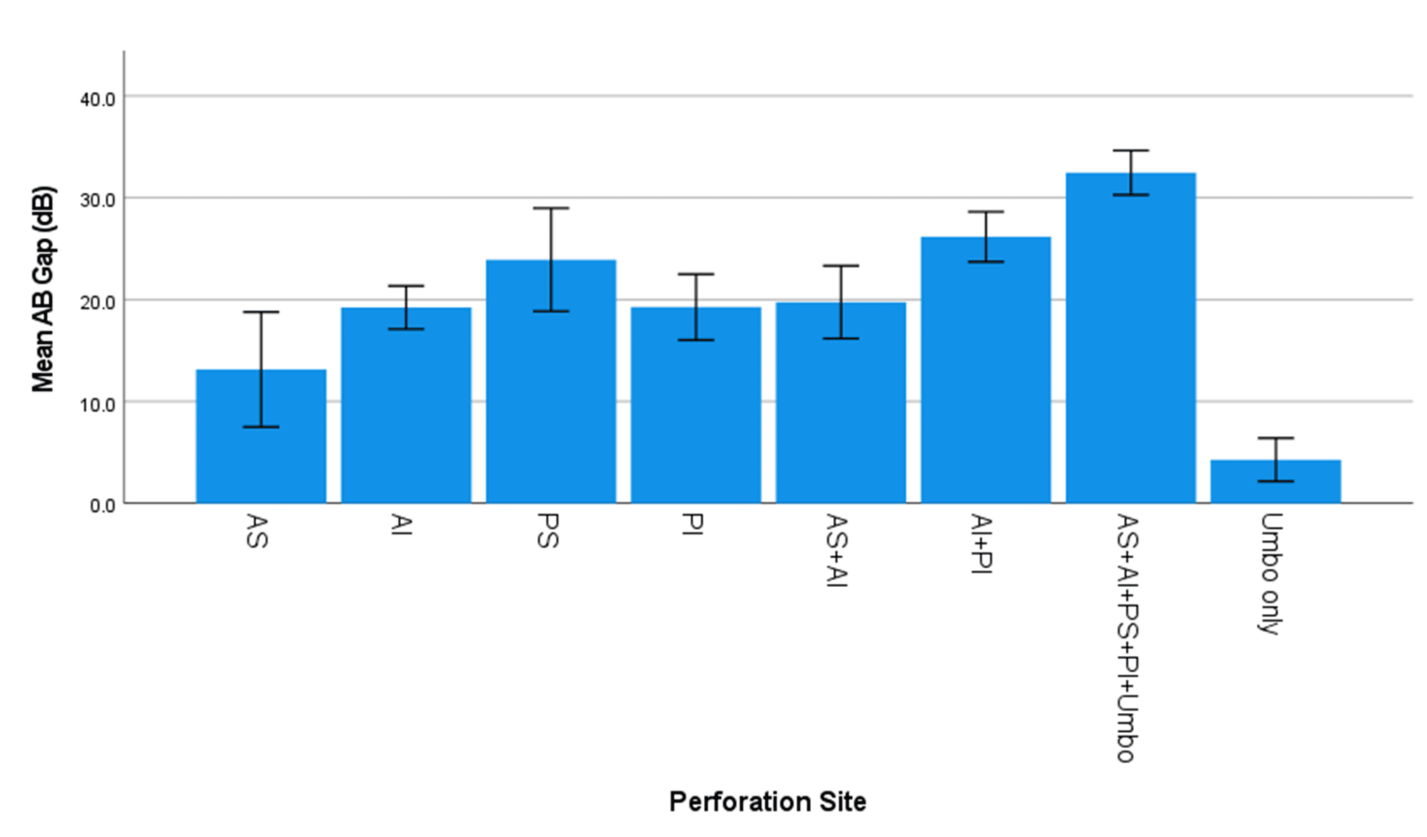
Graph depicting the mean of ABG (dB) by perforation site ABG: air-bone gap

The site of perforation also significantly influenced conductive hearing loss (ABG), with posterior and larger perforations associated with greater hearing loss. Among small perforations, the PI quadrant exhibited the highest ABG (25.33 ± 6.4 dB), while perforations limited to the umbo, which is a small central perforation, had the lowest ABG (4.3 ± 2.8 dB). For medium-sized perforations, AI with PI (AI + PI) exhibited the highest ABG (26.2 ± 6.4 dB), followed by PS quadrant perforations (23.22 ± 8.6 dB). Perforations involving multiple quadrants (AS + AI + PS + PI + Umbo) also resulted in a substantial ABG (24.65 ± 10.1 dB). In the case of large perforations involving all four quadrants and the umbo (AS + AI + PS + PI + Umbo), the most significant ABG was observed (32.47 ± 8.8 dB), while perforations in AI + PI also led to a high ABG (27.6 ± 8.5 dB). These findings indicate that perforation size and location significantly impact hearing loss, with posterior and larger perforations exhibiting greater ABGs (p < 0.001, ANOVA) (Table 2, as illustrated in Figure 1).

**Table 2:** Effect of perforation site on hearing loss (ABG) quadrant wise ABG: air-bone gap; ANOVA: analysis of variance

Site of perforation	Number of perforations		ABG (dB)	p-value
					Small-sized perforation	n	%		
Anterosuperior (AS)	5	2.4	11.3 ± 8.3	<0.001 (ANOVA)
Anteroinferior (AI)	12	5.7	12.36 ± 8.9	
Posterosuperior (PS)	3	1.4	21.8 ± 8.98	
Posteroinferior (PI)	12	5.7	25.33 ± 6.4	
AI + PI	1	0.5	18.3	
Umbo Only	7	3.3	4.3 ± 2.8	
Medium-sized perforation	n	%		
Anterosuperior (AS)	4	1.9	16.25 ± 11.2	<0.001 (ANOVA)
Anteroinferior (AI)	43	20.5	20.94 ± 6.4	
Posterosuperior (PS)	6	2.9	23.22 ± 8.6	
Posteroinferior (PI)	10	4.8	20.63 ± 6.2	
AS + AI	8	3.8	19.04 ± 5.2	
AI + PI	24	11.4	26.2 ± 6.4	
AS + AI + PS + PI + Umbo	4	1.9	24.65 ± 10.1	
Large-sized perforation	n	%		
AS + AI	1	0.5	25.5	<0.001 (ANOVA)
AI + PI	5	2.4	27.6 ± 8.5	
AS + AI + PS + PI + Umbo	65	31.0	32.47 ± 8.8	
Total	210	100		

When TM perforations were divided based on involvement of the handle of the malleus, they were grouped as malleolar and non-malleolar, respectively. Malleolar perforations were associated with greater hearing loss, as compared to non-malleolar (29.26 ± 9.4 vs. 18.25 ± 8.7, p < 0.001) (Table 3).

**Table 3:** Comparison of hearing loss (ABG) between malleolar and non-malleolar perforation ABG: air-bone gap

Groups	Total number of ears	Percentage	ABG (dB)	p-value
Malleolar	110	52.4%	29.26 ± 9.4	<0.001 (t-test)
Non-malleolar	100	47.6%	18.25 ± 8.7	

Effect of perforation size on the relationship between perforation site and ABG

One-way ANOVA demonstrated that perforation size alone significantly influences ABG (p < 0.001). However, when the perforation site was included in a two-way ANOVA model, the main effect of perforation size was no longer significant (F(2,194) = 2.456, p = 0.088), while perforation site remained a strong predictor of ABG (F(7,194) = 3.678, p = 0.001). The interaction between site and size was also not significant (F(6,194) = 1.053, p = 0.392), suggesting that, while perforation size impacts ABG, its effect is secondary to perforation site. This indicates that the relationship between perforation site and ABG remains consistent across different perforation sizes, and size may indirectly contribute to hearing loss depending on the perforation location.

Duration of discharge and ABG

**Figure 2:**
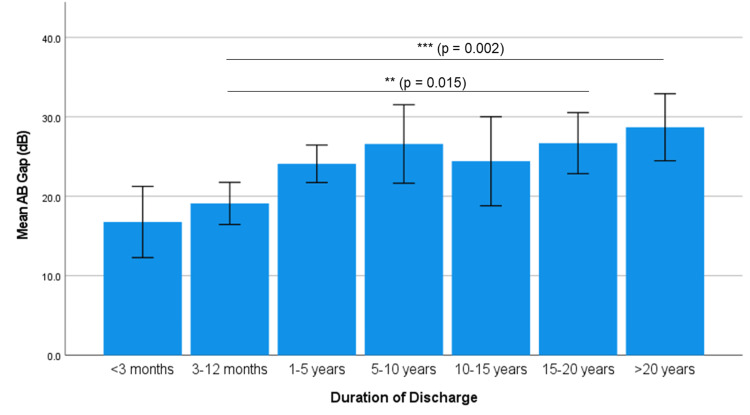
Association of duration of discharge and hearing loss (ABG) ABG: air-bone gap

Shorter discharge durations (<3 months, 3-12 months) showed no significant differences. Patients with >20 years of discharge had the highest ABG (28.69 ± 11.2 dB), while those with <3 months had the lowest (16.77 ± 5.5 dB). There was a significant impact of discharge duration on ABG (p = 0.001), with ABG increasing as discharge duration prolonged. Post hoc Bonferroni analysis showed significant differences between 3-12 months vs. 15-20 years (p = 0.015) and 3-12 months vs. >20 years (p = 0.002). The Spearman's correlation coefficient of 0.323 indicates a weak positive relationship, but the p-value (<0.001) indicates a significant correlation between the duration of discharge and ABG (Table 4 and Figure 2).

**Table 4:** Correlation between the duration of discharge and the hearing loss (ABG) ABG: air-bone gap

Duration of discharge	Number of ears, n (%)	Mean ABG (dB)	Spearman's correlation	
			Correlation coefficient (ρ)	p-value
<3 months	6 (2.9)	16.77 ± 5.5	0.323	<0.001
3-12 months	52 (24.8)	19.11 ± 9.6		
1-5 years	50 (23.8)	24.09 ± 8.3		
5-10 years	24 (11.4)	26.59 ± 12.1		
10-15 years	14 (6.7)	24.41 ± 10.5		
15-20 years	36 (17.1)	26.69 ± 11.5		
>20 years	28 (13.3)	28.69 ± 11.2		

The duration of discharge was self-reported and hence prone to recall bias. Moreover, since longer duration was likely associated with larger perforations and sclerosis, the independent effect of duration on hearing loss was calculated using a multivariable linear regression model, with ABG as the dependent variable, and duration of discharge, presence of sclerosis, and perforation size as independent variables. In multivariable analysis, duration of discharge remained independently associated with greater ABG after adjustment for perforation size and sclerosis (β = 0.19 dB/year, 95% CI: 0.10-0.29, p < 0.001). Both sclerosis (β = 3.70 dB, 95% CI: 1.56-5.83, p = 0.001) and perforation size (β = 9.01 per unit increase, 95% CI: 7.54-10.48, p < 0.001) were also independently significant. The model explained 49.1% of ABG variability (adjusted R² = 0.484), as shown in Table 5.

**Table 5:** Multivariable regression results with hearing loss as dependent variable

Variable	β coefficient	95% CI	p-value
Duration	0.193	0.096-0.290	<0.001
Sclerosis	3.696	1.563-5.830	0.001
Perforation size	9.007	7.535-10.479	<0.001

Radiological findings

In our study, only 177 ears (84%) had undergone X-ray of bilateral mastoids in Schuller's view. The common findings of X-ray are described in Table 6. Within the subset of ears evaluated radiologically, 164 ears (93%) exhibited decreased pneumatization of the mastoid air cell system, while 13 ears (7%) demonstrated adequate pneumatization. Sclerosis of the adjacent mastoid air cell system was noted in 110 ears (62%), while 67 ears (38%) showed no evidence of sclerosis. Ears with normal mastoid pneumatization had the lowest ABG (13.07 ± 3.5 dB), while those with complete sclerosis exhibited the highest (38.3 dB). Ears with decreased pneumatization showed an ABG of 22.47 ± 1.4 dB, which further increased to 26.72 ± 0.9 dB when accompanied by sclerosis. A one-way ANOVA demonstrated a significant effect of X-ray findings on ABG (p < 0.001). Post hoc analysis confirmed that ABG was significantly higher in all diseased conditions (decreased pneumatization, sclerosis, or both) compared to normal (p < 0.05), indicating a strong association between poor mastoid aeration and conductive hearing loss. A chi-square test of independence revealed a significant association between X-ray findings and perforation size (χ² = 9.737, p = 0.042). Normal mastoid pneumatization was more frequently associated with small perforations (5 ears of the total 11 normal pneumatized ears, 45.5%), whereas both decreased pneumatization and sclerosis showed a higher prevalence of large (33.6%, 36 out of 107 ears) and medium-sized (54.2%, 58 out of 107 ears) perforations. Complete sclerosis was found only in two cases, both with large perforations.

**Table 6:** Findings of X-ray (Schuller’s view) and corresponding hearing loss (ABG) AGB: air-bone gap; ANOVA: analysis of variance

X-ray findings (n = 177)	Number of ears	ABG (dB)	p-value	Perforation size			p-value
				Small	Medium	Large	
Normal	11	13.07 ± 3.5	<0.001 (ANOVA)	5	4	2	0.042 Pearson Chi-Square (χ²)
Decreased pneumatization	57	22.47 ± 1.4		12	26	19	
Decreased pneumatization with sclerosis	107	26.72 ± 0.9		13	58	36	
Complete sclerosis	2	38.3		0	0	2	

## Discussion

This study aimed to investigate the relationship between TM perforations - their size, site, and specifically the posterior quadrant - and their impact on hearing loss, as well as the potential influence of clinical features, duration of ear discharge, and radiological findings. Our findings demonstrated that both the size and site of TM perforations significantly influence conductive hearing loss, as measured by the ABG. Additionally, factors such as the duration of discharge and mastoid pneumatization status also appear to play important roles in the extent of hearing impairment observed in the study population.

The site of TM perforation was found to be a strong predictor of conductive hearing loss. In our study, we observed that small central perforations resulted in the most significant hearing loss when located in the PS quadrant, followed by the PI and AI perforations, with the least hearing loss occurring in the AS perforations. This observation aligns with the findings of Nahata et al. [4] and Rana et al. [5], who reported greater hearing loss in perforations located in the PI quadrants (40.07 dB) compared to the AI (17.04 dB) and AS (15 dB) quadrants. However, this contrasts with the studies by Voss et al. [6], Ibekwe et al. [7], and Sood et al. [8], who found no significant relationship between hearing loss and the site of perforation.

Regarding the involvement of the anterior versus posterior regions, our findings showed that, in cases of small central perforations, hearing loss was greater in perforations located in the posterior region compared to the anterior region, although this pattern did not hold for medium-sized perforations. This is consistent with the conclusions of Nahata et al. [4], who reported that posterior perforations (average 39.99 dB) resulted in greater hearing loss compared to central (35.64 dB) or anterior (30.1 dB) perforations, and with Aneesa et al. [9], who also reported greater loss with posterior perforations. In contrast, studies by Sood et al. [8] and Lerut et al. [10] did not find a significant relationship between hearing loss and perforation location in the anterior or posterior quadrants, suggesting that additional anatomical or middle-ear volume factors may modify this effect.

Our study demonstrated a direct correlation between the size of TM perforation and the severity of conductive hearing loss, as reflected by the ABG. As the perforation size increased, the degree of hearing loss also increased: small perforations exhibited an average hearing loss of 13.67 dB, medium-sized perforations 22.13 dB, and large-sized central perforations 34.47 dB, while pinpoint perforations showed a near-normal hearing threshold (mean ABG 4.28 dB, sometimes absent). These findings are consistent with prior research by Voss et al. [6], Ibekwe et al. [7], Sood et al. [8], and Ahmad and Ramani [11], who similarly reported a progressive increase in hearing loss with perforation size, underscoring the need for early identification and treatment of larger defects.

A larger perforation results in a loss of effective vibrating area, reducing sound transmission efficiency and altering the impedance-transforming function of the tympano-ossicular system. Additionally, the loss of ossicular coupling - particularly in the PS quadrant, where the ossicles and round window niche are closely related - contributes to the increased ABG, likely via phase cancellation at the round window and reduction in the effective areal ratio and lever action. These findings support the hypothesis that round window shielding effects and ossicular coupling integrity play a more significant role in hearing loss than the mere loss of TM vibratory area, explaining the disproportionately greater loss seen with posterior and multi-quadrant perforations [12,13].

Perforations involving the malleus were associated with greater hearing loss compared to non-malleolar perforations, consistent with the findings of Gaur et al. [3], Ahmad and Ramani [11], and Pannu et al. [14]. Lerut et al. also reported that perforations involving the umbo can result in an additional hearing loss of approximately 5-6 dB, supporting the concept that the umbo-malleus connection is central to effective sound transmission through the conical TM [10]. Wever and Lawrence explained that, when a perforation involves the umbo, this critical mechanical linkage is disrupted, causing a loss of ossicular coupling and leading to more pronounced hearing impairment than perforations that spare the malleus [12].

This study revealed that, while perforation size independently influences ABG (p < 0.001), its effect becomes statistically insignificant (p = 0.088) when perforation site is included in a two-way ANOVA model. This suggests that the site exerts a more dominant influence on ABG than size, and the interaction between site and size was also non-significant (p = 0.392), indicating that these factors largely contribute additively, rather than synergistically, to conductive hearing loss.

Our findings indicate a significant impact of discharge duration on ABG, with prolonged discharge durations (>15 years) being associated with markedly higher ABGs (p < 0.001). Patients with discharge for more than 20 years exhibited the highest ABG (28.69 ± 11.2 dB), whereas those with discharge durations of less than three months had the lowest (16.77 ± 5.5 dB), and the most notable deterioration occurred after 12 months, with a sharp decline beyond 15 years (ρ = 0.323, weak but significant). These results, in agreement with Ahmad and Ramani [11] and Pannu et al. [14], suggest that persistent inflammation progressively damages the TM and ossicular chain, emphasizing the importance of early diagnosis and timely intervention to prevent cumulative hearing loss. While the duration of discharge had an independent association with ABG, the relationship persisted after rigorous multivariable adjustment for perforation size and sclerosis - the primary structural confounders.

Our study highlights a strong correlation between mastoid air‑cell pneumatization and conductive hearing loss, with the greatest impairment observed in ears showing both decreased pneumatization and sclerosis on radiography. These findings indicate that reduced pneumatization and bony sclerosis - markers of chronic or long-standing otitis media - are associated with increased ABG, likely via reduction of middle ear and mastoid air‑space volume and consequent changes in sound conduction. Voss et al. [6] and Park et al. [15] similarly reported that, for a given perforation size, larger middle ear and mastoid air volumes correlate with less hearing loss, supporting the functional relevance of mastoid pneumatization in mitigating the auditory impact of TM perforations.

We also demonstrated a significant association between mastoid X-ray findings (Schuller’s view) and TM perforation size (χ² = 9.737, p = 0.042), with larger perforations being more frequent in ears with both decreased pneumatization and sclerosis. Patients exhibiting combined sclerosis and decreased pneumatization had a higher likelihood of medium and large perforations (87.8%, 94 of 107 ears), supporting the concept that progressive mastoid disease contributes to more extensive TM defects and greater conductive loss. Complete sclerosis, though rare in our series, was exclusively associated with large perforations, consistent with literature describing mastoid sclerosis as a radiological marker of long-standing disease, while the chi-square test for perforation site and X-ray findings did not show a strong association (p = 0.126).

A key strength of this study lies in its prospective design and relatively large sample size, which enhances reliability and addresses a high-burden condition in a low- and middle-income country setting. Standardized audiometric assessments, quadrant-based perforation classification, and Schüller's view X-ray evaluation enabled an integrated analysis of structural-audiological relationships. Single-investigator measurements minimized inter-observer variability, ensuring consistent data collection and practical applicability of the results.

However, several limitations must be acknowledged. The single-center design, use of clinical (rather than digital or software-based) estimation of perforation size, and exclusion of patients with active disease, cholesteatoma, or sensorineural components may restrict external validity and limit applicability to more complex otologic conditions. The reliance on Schüller’s X-ray, instead of high-resolution computed tomography, constrains the precision of mastoid pneumatization and structural assessment; in addition, subjective estimation of perforation size introduces potential measurement bias, and information on the duration of discharge is vulnerable to recall bias.

Although approximately 30% of patients contributed bilateral ears, potential clustering was formally assessed, and a low intraclass correlation coefficient (ICC < 0.10) suggested minimal impact on the independence assumption; nonetheless, the lack of more advanced modeling for bilateral data weakens statistical rigor compared to mixed-effects approaches. These shortcomings have been acknowledged to provide a balanced interpretation of the findings and to guide cautious extrapolation to broader clinical contexts.

## Conclusions

This study highlights the critical role of TM perforation characteristics, including size, site, and involvement of the malleus, in determining the severity of conductive hearing loss. Our findings confirm that both the site and size of perforations significantly impact hearing thresholds, with perforations in the posterior quadrants - specifically the PS quadrant - contributing to more pronounced hearing impairment. Perforations involving the umbo region, termed malleolar in the study, were associated with higher hearing loss. Hearing loss increased with the increasing size of the TM perforation.

Additionally, prolonged ear discharge was associated with higher hearing loss, highlighting the importance of early diagnosis and intervention in preventing permanent conductive deficits. Radiological evaluations, particularly mastoid X-rays, provided valuable insights into the extent of disease severity, with decreased pneumatization of mastoid air cells and sclerosis associated with higher hearing loss than in their absence. The study was conducted such that clinicians could have an idea of hearing loss and middle ear status from the first visit, allowing a proper assessment of the TM and enabling an individualized treatment plan to be made at the earliest.
